# Interaction between DNMT3B and MYH11 via hypermethylation regulates gastric cancer progression

**DOI:** 10.1186/s12885-021-08653-3

**Published:** 2021-08-12

**Authors:** Jianhua Wang, Ping Xu, Yanping Hao, Tingting Yu, Limin Liu, Yan Song, Yan Li

**Affiliations:** 1 Department of Gastroenterology, The Yancheng Clinical College of Xuzhou Medical University, The First People’s Hospital of Yancheng, Yancheng, 224001 Jiangsu People’s Republic of China; 2 Department of Obstetrics and Gynecology, The Yancheng Clinical College of Xuzhou Medical University, The First People’s Hospital of Yancheng, No. 66, Renmin South Road, Yancheng, 224001 Jiangsu People’s Republic of China

**Keywords:** MYH11, TNFRSF14, DNMT3B, Gastric cancer, Methylation

## Abstract

**Background:**

Gastric cancer (GC) has an unwelcoming prognosis when diagnosed at an advanced stage. The purpose of this study was to examine the expression of myosin heavy chain 11 (MYH11) in GC and mechanisms related.

**Methods:**

The MYH11 expression in GC was investigated via the SangerBox platform. MYH11 expression in GC tissues and cell lines was examined by immunohistochemistry, RT-qPCR, and western blot. The relationship between MYH11 expression and patients’ prognosis was analyzed. The effects of MYH11 on the biological behaviors of GC cells were investigated by gain-of-function experiments. Bioinformatics analysis was used to find genes with relevance to MYH11 expression in GC. The relationship was verified by luciferase and ChIP-qPCR assays, followed by rescue assay validation. The causes of MYH11 downregulation in GC were verified by quantitative methylation-specific PCR. Finally, the effect of MYH11 on tumor growth was examined.

**Results:**

MYH11 was downregulated in GC and predicted poor prognoses. MYH11 reverted the malignant phenotype of GC cells. MYH11 repressed the TNFRSF14 expression by binding to the TNFRSF14 promoter. TNFRSF14 reversed the inhibitory effect of MYH11 on the malignant phenotype of GC cells. The methylation of the MYH11 promoter was elevated in GC, which was correlated with the elevated DNMT3B in GC. Overexpression of DNMT3B repressed transcription of MYH11 by promoting its methylation. Also, MYH11 upregulation inhibited tumor growth.

**Conclusion:**

DNMT3B inhibits MYH11 expression by promoting its DNA methylation, thereby attenuating the repressive effect of MYH11 on the transcriptional of TNFRSF14 and promoting the progression of GC.

**Supplementary Information:**

The online version contains supplementary material available at 10.1186/s12885-021-08653-3.

## Background

Gastric cancer (GC) is expected to affect 26,560 people and cause 11,180 deaths in the United States in 2021 [1]. Even though it is no longer the most frequent malignancy, GC remains the third leading cause of cancer-related death worldwide and the most frequent cancer in the Eastern Asia [2]. The most majority (about 90%) of GC are adenocarcinomas, which stem from the glands of the most superficial layer, or the mucosa, of the stomach [3]. Most of the diagnoses made in the Western world are at late stages during which time the treatment is largely ineffective [4]. Chemotherapy is the regular first-line treatment option for patients with advanced GC and a good performance status, and chemoresistance remains the main challenge in the treatment of GC patients [5]. Therefore, there has been a strong drive to explore molecular mechanisms underlying GC progression to develop new therapies for GC.

Myosin heavy chain 11 (MYH11), encoded by the MYH11 gene, is a smooth muscle myosin of the myosin heavy chain family [6]. It locates on 16P13.11, in 5–10% of de novo acute myeloid leukemia with an inv.(16)(p13q22) or t(16;16) (p13;q22) that formed a fusion gene between CBFB/MYH11 [7]. Mutations of the MYH11 gene have been reported in various forms of cancers, including head and neck cancer [8], non-small-cell lung cancer [9], bladder cancer [10], colon adenocarcinoma [11] as well as GC [12]. However, little is known about its specific role in GC, which awaits to be explored. Moreover, a protein-protein interaction network in a recent work identified MYH11 as a differentially expressed transcription factors in colorectal cancer to be related with metabolism-related genes [13]. Thus, we wondered if the possible role of MYH11 in GC was dependent on its transcription factor role. Furthermore, DNA methylation has a vital part in tumorigenesis through modulating the activation of oncogenes and the depletion of tumor suppressors, while the DNA methylation of MYH11 promoter was revealed to be engaged in the progression of GC [14]. This study, as a consequence, investigated the expression of MYH11 in GC and analyzed the association of MYH11 expression with the DNA methylation in GC. In addition, the possible role of MYH11 in GC was further investigated in vitro and in vivo. Moreover, we also set to determine the downstream target of MYH11 in GC. The answer to these questions might offer a novel strategy for GC diagnostic strategy and treatment.

## Methods

### Patients

GC tumor tissues and their adjacent tissues were collected from 40 patients who underwent surgery at the First People’s Hospital of Yancheng between February 2014 and January 2015. All patients had a complete medical history, did not have other malignancies, and did not receive preoperative radiotherapy or chemotherapy. The tissues were stored at − 80 °C immediately after surgery. The study protocol was approved by the Ethics Committee of the First People’s Hospital of Yancheng. Written informed consent was acquired from all of the patients.

### Immunohistochemistry (IHC)

The tissues were fixed using 10% formalin, then embedded in paraffin and cut into 4-μm sections. Xylene and alcohol were used for dewaxing and rehydration. Endogenous peroxidase activity was blocked by using hydrogen peroxide (30%), and the sections were boiled for approximately 3 min with citrate buffer (pH = 6.0). Next, normal goat serum was used to incubate the sections to reduce non-specific binding. The tissue sections were incubated (4 °C, 12 h) with primary antibodies to MYH11 (1:250, ab133567, Abcam, Cambridge, UK), TNFRSF14 (1:250, #PA5–20237, Thermo Fisher Scientific Inc., Waltham, MA, USA), and Ki67 (1:200, ab16667, Abcam) and with secondary antibody to IgG (1:1000, ab6721, Abcam) for 2 h at ambient temperature. Finally, the sections were stained with 3,3-diaminobenzidine for 60 s, stained with hematoxylin for 2 min, and sealed with neutral resin. A fluorescence photomicroscope was used to acquire images (Nikon Instruments Inc., Melville, NY, USA), and the positivity rate was measured by Image J software.

The expression was assessed based on staining intensity and percentage of positive cells by two pathologists who were unaware of the grouping. The intensity of staining was scored as 0 (negative), 1 (weakly negative), 2 (weakly positive), and 3 (strongly positive). The percentage scores of positive cells in each single field of view were 1 (0–25%), 2 (26–50%), 3 (51–75%), and 4 (76–100%). We multiplied the above two scores together to give a final score of 0 to 12.

### RNA extraction and RT-qPCR

Total RNA was isolated from tissues and cells with the help of TRIzol reagent (Takara Holdings Inc., Kyoto, Japan). The concentration and purity of RNA samples were measured by Nanodrop 2000 (Thermo Fisher Scientific). Total RNA was reversely transcribed to cDNA using the PrimeScript RT kit (Takara). Then, RT-qPCR was performed using TB Green Premix Ex Taq II (Takara) and ABI Prism 7500 Sequence Detection System (Applied Biosystems, Inc., Foster City, CA, USA). Glyceraldehyde-3-phosphate dehydrogenase (GAPDH) was selected as a housekeeping gene, and the comparative cycle threshold (Ct) method (2^-ΔΔCt^) was used to analyze differences in each transcript. The primer sequences for the tested genes are provided in Table 1.

**Table 1:** Primer used for RT-qPCR

Gene	Forward primer (5′-3′)	Reverse primer (5′-3′)
MYH11	GTCCAGGAGATGAGGCAGAAAC	GTCTGCGTTCTCTTTCTCCAGC
TNFRSF14	TTCTCTCAGGGAGCCTCGTCAT	CTCACCTTCTGCCTCCTGTCTT
DNMT3B	TAACAACGGCAAAGACCGAGGG	TCCTGCCACAAGACAAACAGCC
GAPDH	GTCTCCTCTGACTTCAACAGCG	ACCACCCTGTTGCTGTAGCCAA

Note: MYH11, myosin heavy chain 11; TNFRSF14/HVEM, herpes virus entry mediator; DNMT3B, DNA methyltransferase 3b; GAPDH, glyceraldehyde-3-phosphate dehydrogenase

### Cell culture and treatment

GES-1 (CL-0563) and GC cell line MKN-7 (CL-0574) were purchased from Procell (Wuhan, Hubei, China). GC cells AGS (CRL-1739), SNU-16 (CRL-5974), NCI-N87 (CRL-5822) were from ATCC (Manassas, VA, USA). All cells were cultivated at 37 °C in Roswell Park Memorial Institute-1640 complete medium (PM150110B, Procell) plus 10% FBS with 1% penicillin/streptomycin at 5% CO_2_.

The overexpressed (oe) DNA plasmids oe-MYH11, oe-TNFRSF14, oe-DNMT3B and control oe-negative control (NC) used for cell transfection were from VectorBuilder (Guangzhou, Guangdong, China). Lipofectamine 2000 (Thermo Fisher) was used for all transfections. Dimethylsulfoxide (DMSO) and DNA methylation inhibitor 5-aZa-CDR (CAS No. 2353-33-5) were purchased from Sigma-Aldrich Chemical Company (St Louis, MO, USA). The GC cells were treated with 5-aZa-CDR dissolved in DMSO at 5 μM for 24 h for DNA demethylation [15].

### Western blot

The cells were lysed using radio immunoprecipitation assay lysis buffer on ice (Beyotime, Shanghai, China). Proteins were quantified by bicinchoninic acid assay kit analysis (Thermo Fisher), separated by 10% sodium dodecyl sulfate-polyacrylamide gel electrophoresis, and transferred to polyvinylidene difluoride membranes (Millipore Corp, Billerica, MA, USA). The membranes were sealed using 5% skim milk powder and then incubated overnight at 4 °C with the primary antibody and with the secondary antibody for 2 h at ambient temperature. Finally, the protein bands were visualized by the Enhanced Chemiluminescent Western Blotting Substrate Kit (Abcam). Relative expression of proteins was analyzed using Quantity One software with GAPDH as an internal reference. All antibodies used in the present study is exhibited in Table 2.

**Table 2:** Antibodies used for western blot

Antibodies	Dilution	Catalog number	Manufacturers
Primary antibodies			
MYH11	1/1000	ab133567	Abcam
TNFRSF14	1/500	#PA5–20237	Thermo Fisher Scientific
GAPDH	1/1000	#2118	Cell signaling technology
Secondary antibody			
IgG	1/2000	ab6721	Abcam

Note: MYH11, myosin heavy chain 11; GAPDH, glyceraldehyde-3-phosphate dehydrogenase; MYH11, myosin heavy chain 11

### Quantitative methylation-specific PCR (qMSP)

Human genomic DNA was obtained from the tissues or cells using a genomic DNA kit (TIANGEN Biotech, Beijing, China) and then modified with bisulfite using a DNA methylation kit (Zymo Research, Orange, CA, USA). PCR products were evaluated by using qPCR. The MSP primers for the MYH11 promoter CpG island were designed using MethPrimer (http://www.urogene.org/cgi-bin/methprimer/methprimer.cgi) with the following primer sequences: left methylated-specific primer: CGGGGAGTAGGAAGGTTATTC; right methylated-specific primer: ACAACCCAAAAAAAACAAACG. left unmethylated-specific primer: GTTGGGGAGTAGGAAGGTTATTT; right unmethylated-specific primer: AACAACCCAAAAAAAACAAACAC.

### Cell counting kit-8 (CCK-8)

Changes in the proliferative capacity of cells were detected using a CCK-8 kit (Dojindo, Kumamoto, Japan). The GC cells were plated in 96-well plates at 3000 cells/well and incubated for the indicated times (0 h, 24 h, 48 h, 72 h), and 10 μL CCK-8 reagent was added directly to the culture medium. The cells were incubated at 37 °C for 2 h, and the optical density (OD) value was measured at 450 nm using a microplate reader (BioTeke Corporation, Wuxi, Jiangsu, China).

### Colony formation assay

The cells were plated into a 6-well plate at a density of 1000 cells per well and then incubated for 2 weeks at 37 °C in 5% CO_2_. Then, the cells were fixed with 4% paraformaldehyde for 20 min and stained with 0.5% crystal violet solution for 20 min. The colonies were then counted and analyzed.

### TUNEL assay

YF®488 TUNEL Assay Apoptosis Detection Kits (Yuhengbio, Suzhou, Jiangsu, China) were used to evaluate the apoptosis rate of GC cells. In brief, the cells were fixed with 4% paraformaldehyde solution (pH = 7.4) for 30 min and then treated with 0.2% TritonX-100 for 20 min for permeabilization. After incubation with 100 μL TUNEL equilibration buffer per well for 5 min, the cells were reacted with 50 μL TUNEL reaction mixture for 60 min under light-proof conditions. Nuclei were stained for ten minutes using 2 μg/mL 4′,6-diamidino-2-phenylindole (DAPI) solution. The cells were then viewed by a fluorescence microscope, and the apoptotic cells were labeled with bright green fluorescence. Apoptosis rate was calculated as the ratio of TUNEL-positive cells to the total number of DAPI-positive cells in five randomly selected regions.

### Wound healing assay

Wound healing assays were performed to determine the migratory capacity of the cells. The GC cells were plated in 6-well plates at 1 × 10^4^ cells and allowed to grow to 80–90% confluence. Then, a thin wound was created using a sterile pipette tip (200 μL). The gap between the wound edges was imaged at the time points of 0 and 48 h. Wound healing rate (%) was evaluated by comparing the difference in the wound width by ImageJ software.

### Transwell assay

Cell invasion was assessed using 24-well chambers (8.0 μm, Corning Glass Works, Corning, N.Y., USA) pre-coated with Matrigel (BD Biosciences, San Jose, CA, USA). The GC cells (1 × 10^5^) dispersed in 200 μL serum-free medium were placed into the apical chamber, and 600 μL medium containing 10% FBS was added in the basolateral chamber. The cells invaded into the basolateral chamber were fixed with 4% paraformaldehyde after 24 h and then treated with 0.5% crystal violet solution. Observations were made under a microscope, and five fields of view were randomly selected to count the number of invaded cells.

### Luciferase reporter assay

A luciferase reporter assay was used to investigate the effect of MYH11 on the transcriptional activity of the TNFRSF14 promoter. The potential binding sites between MYH11 and the TNFRSF14 promoter were obtained from hTFtarget (http://bioinfo.life.hust.edu.cn/hTFtarget/#!/). The TNFRSF14 promoter sequence containing the binding site was inserted into the pGL3-basic vector (Promega Corporation, Madison, WI, USA) to construct a luciferase reporter vector. The above vector plasmids were co-transfected with oe-NC or oe-MYH11 into 293 T cells (ATCC) using Lipofectamine 2000, respectively. After 48 h of transfection, luciferase assays were performed using the Dual Luciferase Reporter Gene Assay System kit (Promega).

### Chromatin immunoprecipitation (ChIP)

Protein-DNA interaction was detected using the Pierce™ Agarose ChIP Kit (Thermo Fisher Scientific). Briefly, the cells are cross-linked using formaldehyde solution, and chromatin was sheared by enzymatic digestion with micrococcal nuclease (Thermo Fisher). The chromatin was incubated overnight at 4 °C with antibodies to MYH11, DNMT3A, DNMT3B and 20 μL protein A/G plus agarose at 4 °C, with antibody to IgG as a control. All antibodies were supplied by Thermo Fisher. After immunoprecipitation elution and DNA recovery, the purified DNA was subjected to qPCR.

### Animal experiments

This study was approved by the Animal Care and Ethics Committee of the First People’s Hospital of Yancheng and carried out in compliance with the Animals in Research: Reporting In vivo Experiments (ARRIVE) guidelines. All animal procedures were in accordance with the Guide for the Care and Use of Laboratory animals published by the US National Institutes of Health. Eight 4-week-old male BALB/c nude mice were from Beijing Vital River Laboratory Animal Technology Co., Ltd. (Beijing, China). MKN-7 cells (1 × 10^7^) stably transfected with oe-NC or oe-MYH11 were injected subcutaneously into the right ventral side of nude mice, 4 nude mice per group. The tumor volume was measured weekly with calipers. Tumor volume was calculated as (length × width^2^)/2. Finally, the mice were euthanized, and the weight of the tumor tissues was examined. The gene expression in tumor tissues was detected by immunohistochemical staining.

### Statistical analysis

All values are expressed as the mean ± SD. Each assay was performed at least three times. Statistical significance was analyzed by *t* tests and one-way or two-way ANOVA and Tukey’s multiple range tests. The threshold of significance was selected as a *p* value < 0.05. Fisher’s exact test was used to test for the clinicopathologic parameters of the patients, and log-rank test was used to analyze the survival rate of the patients. Quantitative data analysis was performed in Graph Pad Prism 8.0 (GraphPad Software, Inc., La Jolla, CA, USA).

## Results

### Downregulation of MYH11 in GC is associated with poor prognosis of patients

**Fig. 1:**
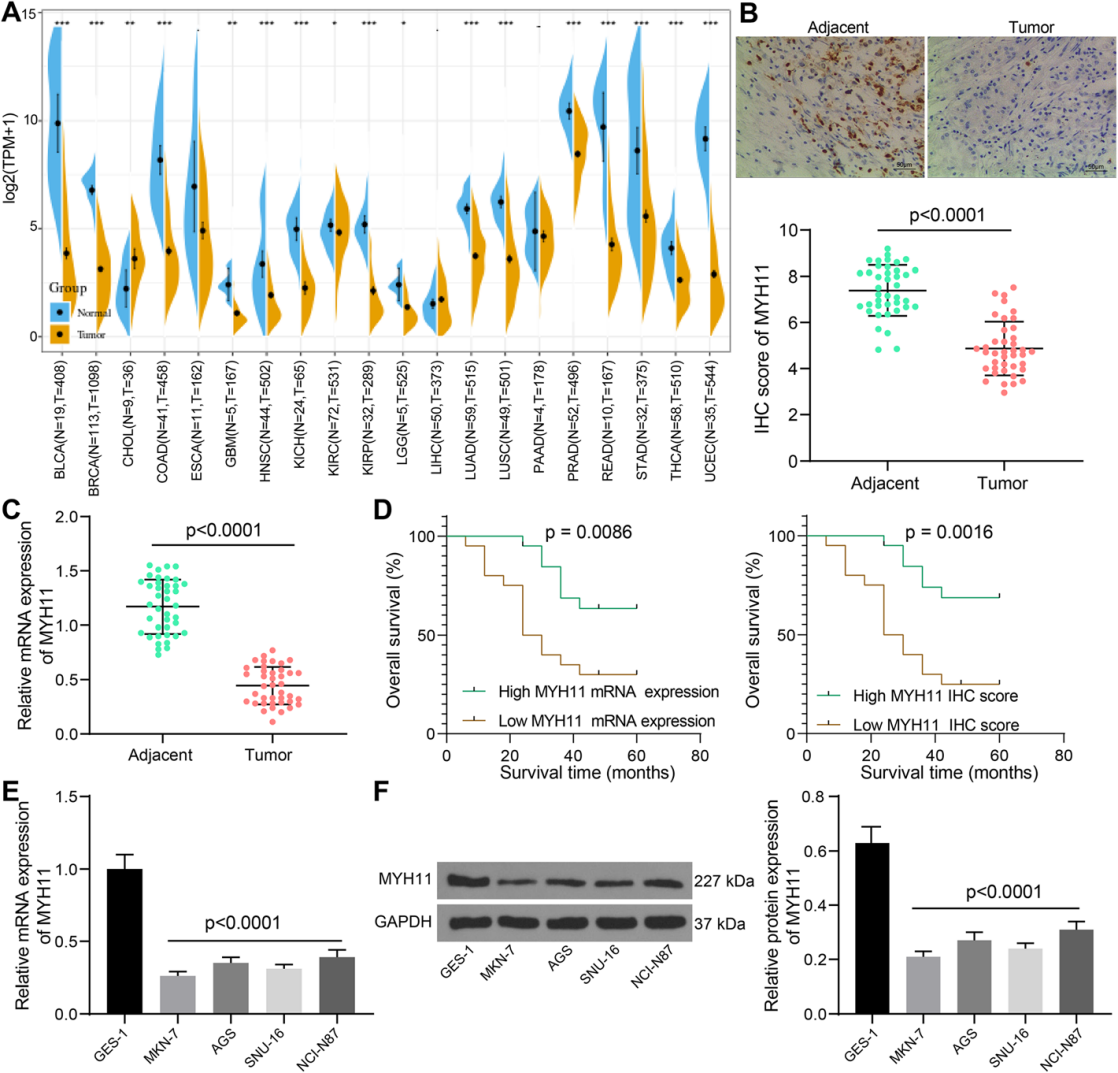
MYH11 expresses poorly in GC and acts as an indicator for good prognosis. **A**, MYH11 expression in cancers examined by the SangerBox (http://sangerbox.com/Tool); **B**, immunohistochemical detection of MYH11 expression in the collected tumor tissues and their adjacent tissues; **C**, MYH11 expression in collected tumor tissues and their adjacent tissues by RT-qPCR; **D**, relationship between MYH11 expression in tumor tissues and five-year survival rate of patients examined by Log-rank test; **E**, MYH11 expression in gastric mucosal epithelial cell line and GC cells by RT-qPCR; **F**, MYH11 expression in gastric mucosal epithelial cell line and GC cells by western blot. Each assay was performed at least three times. Statistical significance was analyzed by *t* tests (panels B and C) and one-way ANOVA (panels E and F) and Tukey’s multiple range tests

We performed a pan-cancer analysis in SangerBox (http://sangerbox.com/Tool) and observed a significant downregulation of MYH11 in most cancers, such as GC (Fig. 1A). We then detected the expression of MYH11 in the tumor tissues of GC patients and their adjacent normal tissues by IHC (Fig. 1B). We found that IHC scores of MYH11 were significantly lower in tumor tissues than that in adjacent tissues. The downregulation of MYH11 in tumor tissues was further substantiated by RT-qPCR assay (Fig. 1C). The patients were divided into MYH11 mRNA high expression group (*n* = 20) and MYH11 mRNA low expression group (n = 20) according to the median value of MYH11 mRNA expression in tumor tissues. We then divided the patients into MYH11 high IHC score group (n = 20) and MYH11 low IHC score group (n = 20) based on the median MYH11 IHC score in the tumor tissues. The five-year survival rate of patients with high MYH11 expression was significantly higher than that of patients with low MYH11 expression (Fig. 1D). By analyzing the clinicopathologic parameters of the patients, we observed that the poor expression of MYH11 also correlated with the tumor size, TNM stage, and lymph node metastasis of the patients (Table 3).

**Table 3:** Relationship between clinicopathologic parameters and the expression of MYH11 in GC

Parameters		Total cases	MYH11 mRNA expression		*p* value	MYH11 IHC score		*p* value
		(*n* = 40)	High (*n* = 20)	Low (*n* = 20)		High (*n* = 20)	Low (*n* = 20)	
Sex	Male	26	12	14	0.7411	11	15	0.3203
	Female	14	8	6		9	5	
Age	≧ 60	21	12	9	0.5273	13	8	0.2049
(year)	< 60	19	8	11		7	12	
Tumor size	≧ 3	25	9	16	*0.0484	7	18	***0.0008
(cm)	< 3	15	11	4		13	2	
TNM stage	I + II	17	13	4	**0.0095	14	3	**0.0011
	III + IV	23	7	16		6	17	
Tumor site	Upper GC	22	13	9	0.3406	10	12	0.7512
	Distal end GC	18	7	11		10	8	
Lymph node metastasis	Positive	25	9	16	*0.0484	7	18	***0.0008
	Negative	15	11	4		13	2	

Note: Fisher’s exact test was used to test for the clinicopathologic parameters of the patients. *p < 0.05, **p < 0.01, ***p < 0.001. MYH11, myosin heavy chain 11; GC, gastric cancer; TNM, tumor, node, metastases; IHC, immunohistochemistry

In in vitro experiments, we detected the expression of MYH11 in GES-1 and GC cell lines MKN-7, AGS, SNU-16, and NCI-N87 by RT-qPCR and western blot (Fig. 1E, F). The expression of MYH11 was generally reduced in GC cell lines, and MKN-7 and SNU-16 cells with the most significantly reduced MYH11 expression were selected for subsequent cell experiments.

### Overexpression of MYH11 inhibits proliferation, migration, invasion of GC cells, and promotes apoptosis

**Fig. 2:**
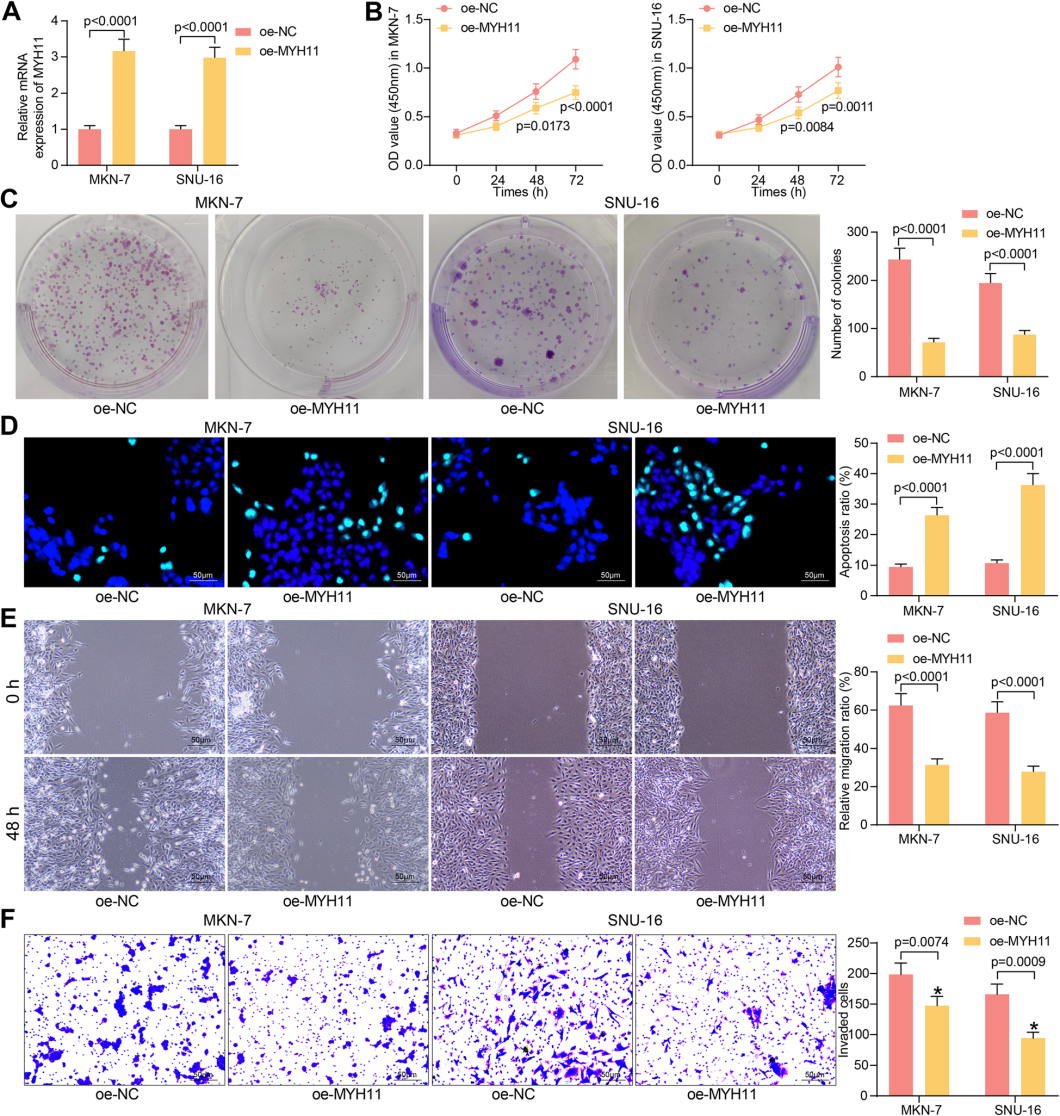
MYH11 reverts the malignant phenotype of GC cells. **A**, transfection efficiency of oe-MYH11 by RT-qPCR; **B**, cell proliferation ability examined by CCK8 assay; **C**, cell colony formation detected by the colony formation assay; **D**, apoptosis rate examined by TUNEL assay; **E**, cell migration examined by wound healing assay; **F**, cell invasion ability assessed by Transwell assays. Each assay was performed at least three times. Statistical significance was analyzed by two-way ANOVA (panels A-F) and Tukey’s multiple range tests

To investigate the effect of abnormally reduced MYH11 on GC cell function, we transfected the overexpression plasmid oe-MYH11 and its control oe-NC into GC cells, and effective transfection was measured by RT-qPCR (Fig. 2A). Overexpression of MYH11 significantly inhibited the growth of GC cells by the CCK8 assay (Fig. 2B). The colony formation assay demonstrated the inhibitory ability of MYH11 on GC cell colony formation (Fig. 2C). We also observed a significant increase in the apoptosis rate of GC cells after overexpression of MYH11 by TUNEL assay (Fig. 2D). Wound healing assay and Transwell assay were used to detect changes in the migration and invasion of cells (Fig. 2E, F). Overexpression of MYH11 significantly repressed the aggressiveness of GC cells.

### MYH11 correlates to TNFRSF14 expression in GC

**Fig. 3:**
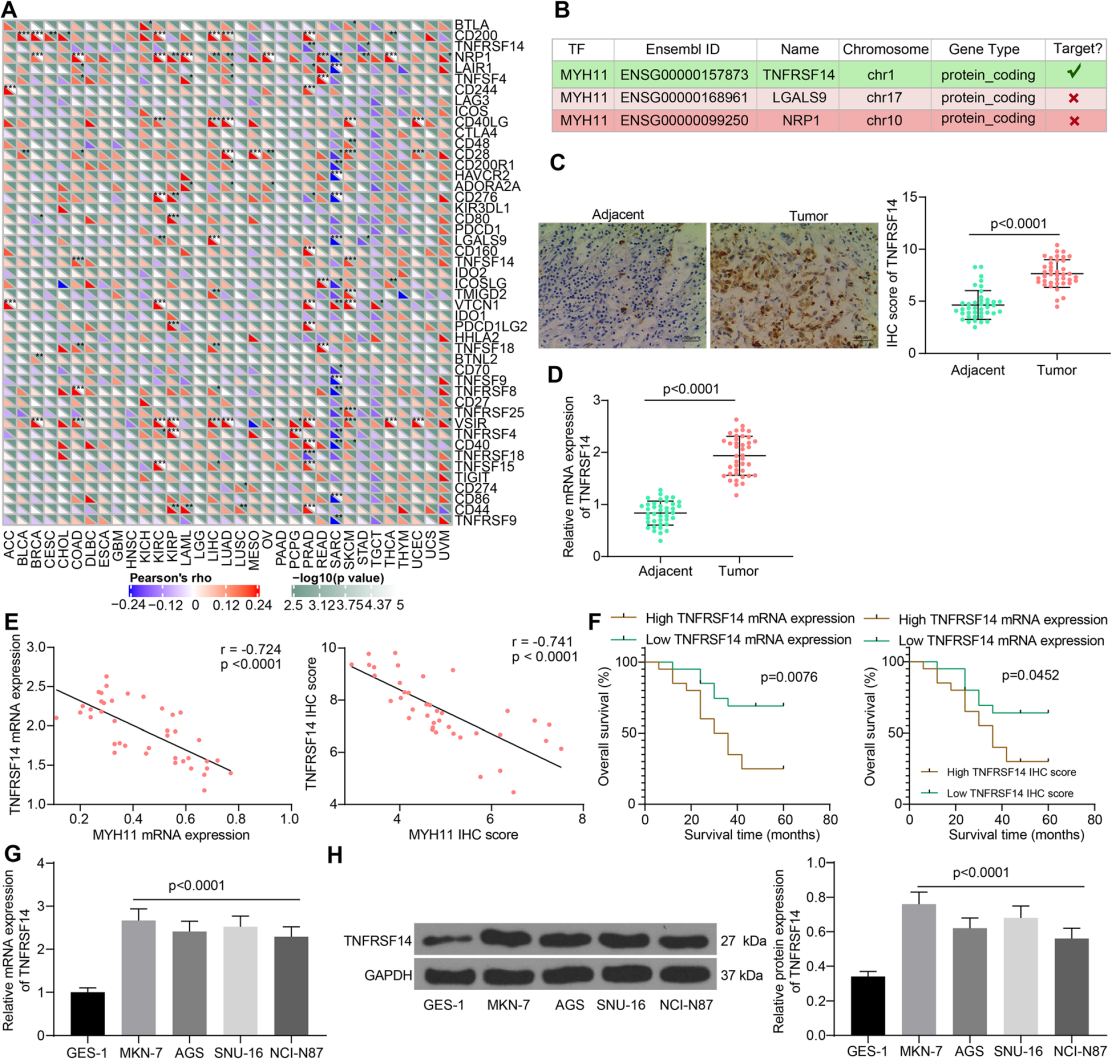
TNFRSF14 expression correlates with MYH11 expression in GC. **A**, factors with significant correlation with MYH11 expression in cancers; **B**, the possible interacting relation between MYH11 and TNFRSF14; **C**, immunohistochemical detection of TNFRSF14 expression in tumor tissues and their adjacent tissues; **D**, TNFRSF14 expression in tumor tissues and their adjacent tissues by RT-qPCR; **E**, correlation between TNFRSF14 expression and MYH11 expression in tumor tissues analyzed by Pearson’s correlation analysis (r = −0.7240, *p* < 0.001); **F**, five-year survival analysis of patients with different TNFRSF14 expression analyzed using Log-rank test; **G**, TNFRSF14 mRNA expression in GES-1 cells and GC cells by RT-qPCR; **H**, TNFRSF14 protein expression in GES-1 cells and GC cells by western blot. Each assay was performed at least three times. Statistical significance was analyzed by paired *t* test (panels C and D) or one-way ANOVA (panels G and H) and Tukey’s multiple range tests

To investigate the mechanism by which MYH11 plays a tumor repressive role in GC, we investigated the genes with correlation with MYH11 expression in TCGA database by SangerBox. Three genes, including TNFRSF14, NRP1, and LGALS9 were correlated with MYH11 expression in GC (Fig. 3A). Subsequently, we predicted through hTFtarget that MYH11 has a potential targeting relationship with TNFRSF14, while no potential targeting relationship with NRP1 and LGALS9 (Fig. 3B).

We first detected the expression of TNFRSF14 in GC tumor tissues and their adjacent tissues by IHC (Fig. 3C). The expression of TNFRSF14 was enhanced in tumor tissues relative to adjacent tissues. The mRNA expression of TNFRSF14 was detected by RT-qPCR, and we observed that its expression was significantly elevated in tumor tissues (Fig. 3D). The expression of TNFRSF14 in tumor tissues was significantly and negatively correlated with the expression of MYH11 (Fig. 3E). The relationship between TNFRSF14 expression and patients’ survival was analyzed, and the results showed that the survival rate of patients with low TNFRSF14 expression was significantly higher than that of patients with high expression (Fig. 3F). The high expression of TNFRSF14 was associated with tumor size, TNM stage and lymph node metastasis (Table 4). Meanwhile, the expression of TNFRSF14 was also significantly elevated in GC cells by RT-qPCR and western blot (Fig. 3G, H).

**Table 4:** Relationship between clinicopathologic parameters and the expression of TNFRSF14 in GC

Parameters		Total cases	TNFRSF14 mRNA expression		*p* value	TNFRSF14 IHC score		*p* value
		(*n* = 40)	High (*n* = 20)	Low (*n* = 20)		High (*n* = 20)	Low (*n* = 20)	
Sex	Male	26	16	10	0.0958	13	13	> 0.9999
	Female	14	4	10		7	7	
Age	≧60	21	8	13	0.2049	9	12	0.5273
(year)	< 60	19	12	7		11	8	
Tumor size	≧3	25	17	8	**0.0079	16	9	*0.0484
(cm)	< 3	15	3	12		4	11	
TNM stage	I + II	17	3	14	**0.0011	2	15	***0.0004
	III + IV	23	17	6		18	5	
Tumor site	Upper GC	22	11	11	> 0.9999	14	8	0.1110
	Distal end GC	18	9	9		6	12	
Lymph node metastasis	Positive	25	17	8	**0.0079	19	6	***0.0002
	Negative	15	3	12		1	14	

Note: Fisher’s exact test was used to test for the clinicopathologic parameters of the patients. *p < 0.05, **p < 0.01, ***p < 0.001. TNFRSF14/HVEM, herpes virus entry mediator; GC, gastric cancer; TNM, tumor, node, metastases; IHC, immunohistochemistry

### MYH11 transcriptionally represses the expression of TNFRSF14 by binding to its promoter

**Fig. 4:**
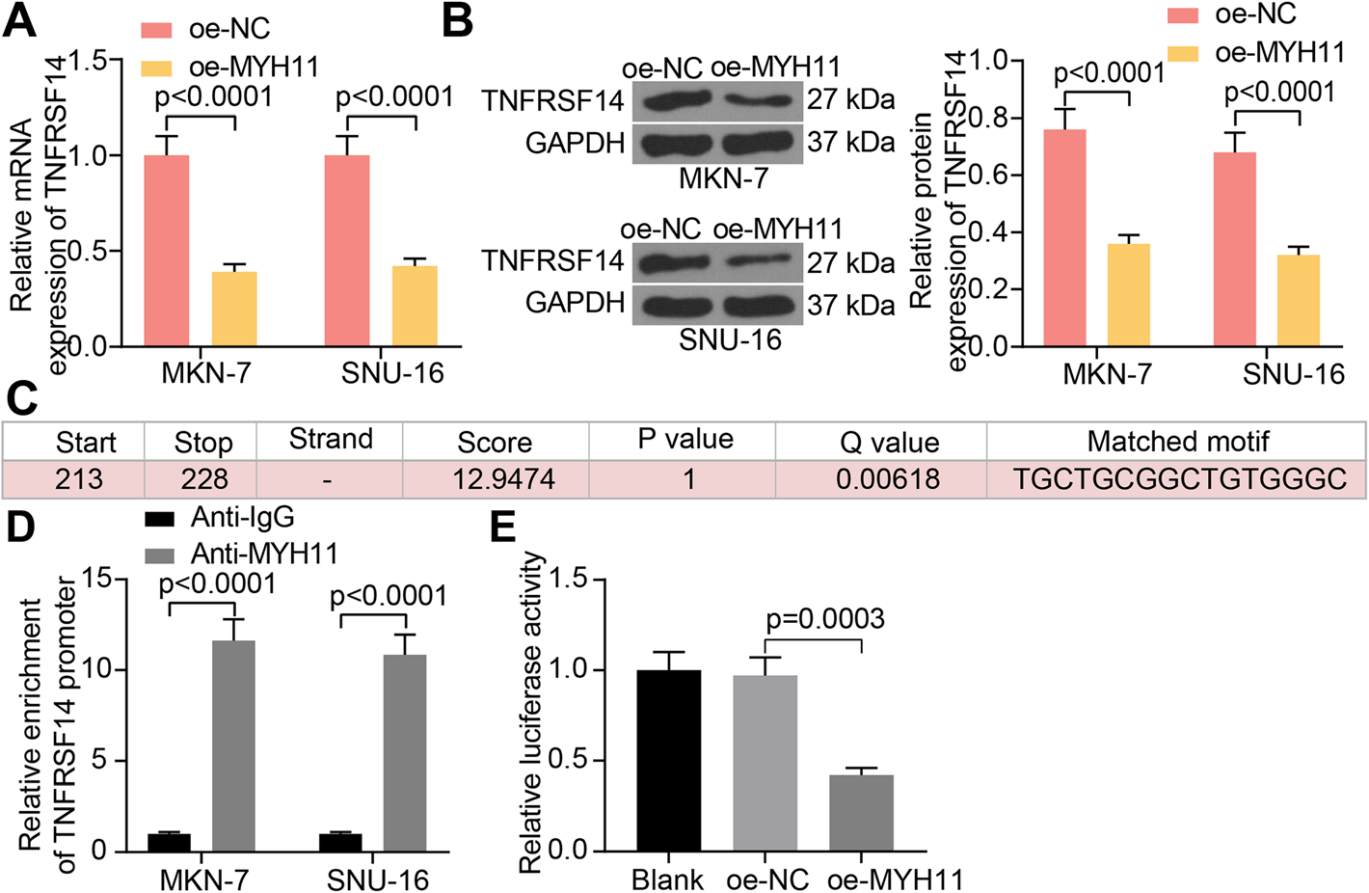
MYH11 interacts with TNFRSF14 by direct binding in GC cells. **A**, TNFRSF14 expression in GC cells in response to oe-MYH11 or oe-NC by RT-qPCR; **B**, TNFRSF14 expression in GC cells in response to oe-MYH11 or oe-NC by western blot; **C**, the binding sites between MYH11 and TNFRSF14 promoter; **D**, the enrichment ability of MYH11 on TNFRSF14 promoter examined by ChIP-qPCR; **E**, the transcriptional activity of TNFRSF14 promoter detected by a luciferase report assay. Each assay was performed at least three times. Statistical significance was analyzed by one-way (panel E) or two-way ANOVA (panels A, B and D) and Tukey’s multiple range tests

The effect of overexpression of MYH11 on TNFRSF14 expression in GC cells was examined by RT-qPCR and western blot, and we observed that overexpression of MYH11 significantly inhibited TNFRSF14 expression (Fig. 4A, B). To detect the way MYH11 regulates TNFRSF14 expression, we obtained TNFRSF14 promoter sites from hTFtarget with a potential binding relationship with MYH11 (Fig. 4C). ChIP-qPCR experiments were performed in GC cell lines to detect the enrichment ability of MYH11 for the TNFRSF14 promoter (Fig. 4D). Anti-MYH11 significantly enriched the TNFRSF14 promoter sequence compared to anti-IgG. A luciferase reporter assay was performed to examine the effect of MYH11 on the TNFRSF14 promoter transcriptional activity (Fig. 4E). Overexpression of MYH11 significantly inhibited the promoter transcriptional activity of TNFRSF14.

### Overexpression of TNFRSF14 reverses the inhibitory effect of MYH11 on GC cells

**Fig. 5:**
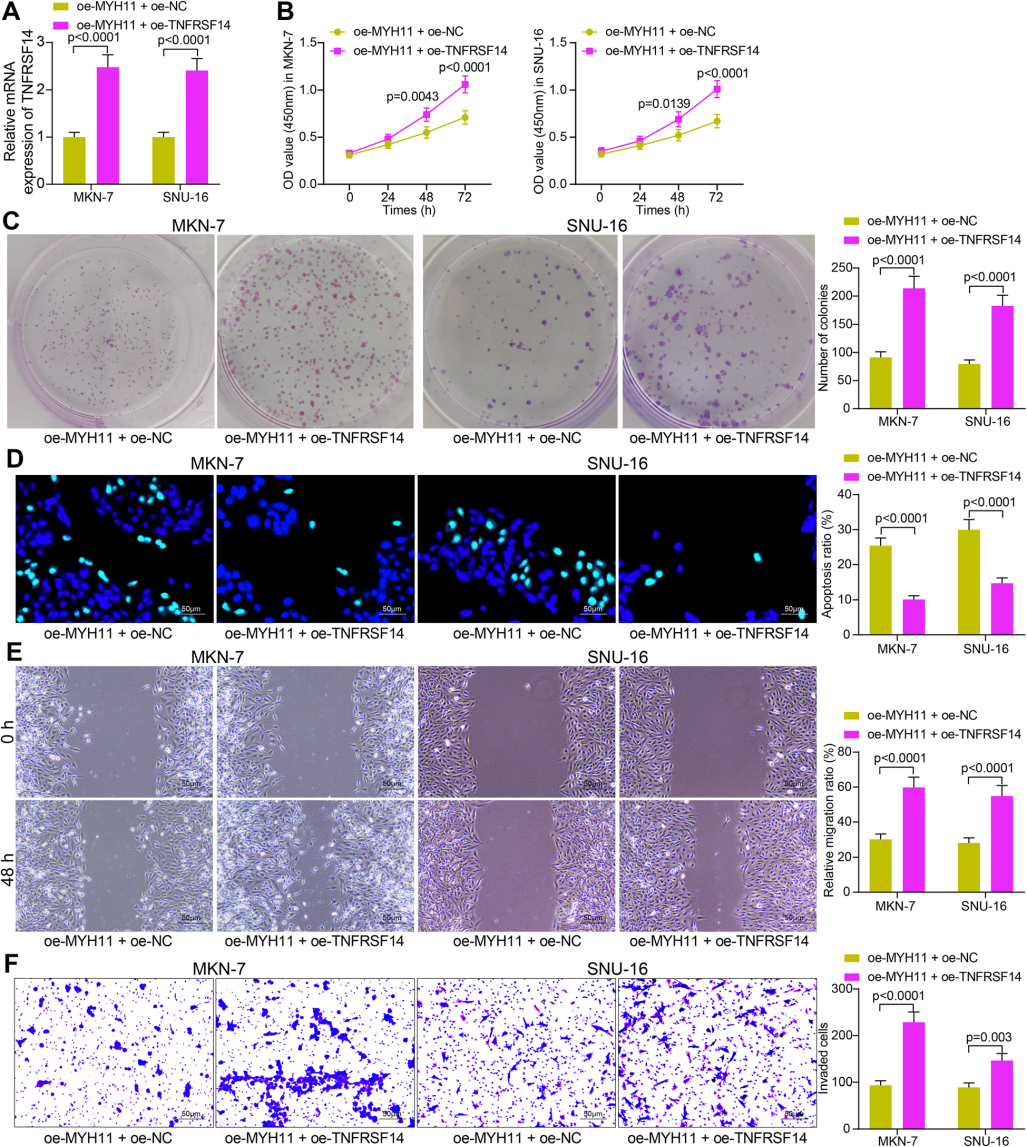
TNFRSF14 overexpression inhibits the repressive effects of oe-MYH11 on GC cell malignant phenotype. GC cells were co-transfected with oe-MYH11 + oe-TNFRSF14. **A**, efficiency of co-transfection by RT-qPCR; **B**, cell proliferation ability examined by CCK8 assay; **C**, cell colony formation detected by the colony formation assay; **D**, apoptosis rate examined by TUNEL assay; **E**, cell migration ability evaluated by wound healing assay; **F**, cell invasion ability assessed by Transwell assays. Each assay was performed at least three times. Statistical significance was analyzed by two-way ANOVA (panels A-F) and Tukey’s multiple range tests

To demonstrate that TNFRSF14 is a downstream functional target of MYH11, we transfected the TNFRSF14 overexpression plasmid oe-TNFRSF14 and the control oe-NC into GC cells overexpressing MYH11. Effective transfection was measured by RT-qPCR (Fig. 5A). CCK8 analysis showed that overexpression of TNFRSF14 significantly enhanced the proliferation of GC cells (Fig. 5B). Also, overexpression of TNFRSF14 promoted the colony formation of GC cells (Fig. 5C). TUNEL assay showed that the apoptosis rate was significantly reduced after overexpression of TNFRSF14 (Fig. 5D). As for changes in the migration and invasion of cells, overexpression of TNFRSF14 significantly reversed the inhibitory effect of MYH11 on the GC cell migration and invasion (Fig. 5E, F).

### Elevated methylation level of MYH11 promoter is identified in GC

**Fig. 6:**
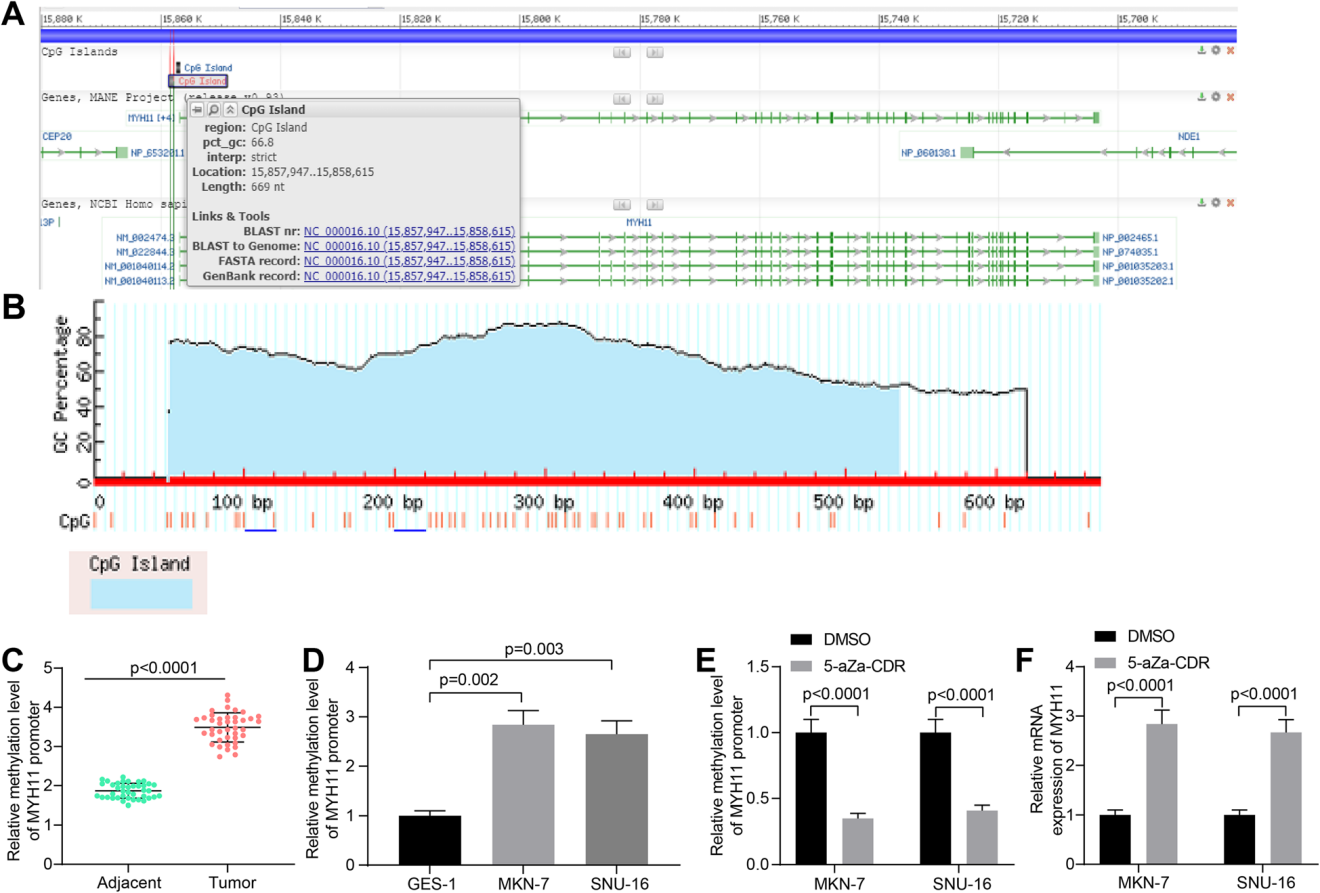
MYH11 promoter is methylated in GC. **A**, the MYH11 promoter CpG island was queried in NCBI; **B**, validation of CpG island sequences; **C**, the methylation level of MYH11 promoter in GC tumor tissues and their adjacent tissues examined by qMSP; **D**, the methylation level of MYH11 promoter in GSE-1 cells and GC cells examined by qMSP; **E**, the effect of 5-aZa-CDR treatment on the methylation level of MYH11 promoter in GC cells examined by qMSP; **F**, the effects of 5-aZa-CDR treatment on MYH11 expression in GC cells by RT-qPCR. Each assay was performed at least three times. Statistical significance was analyzed by paired *t* test (panel C), one-way (panel D) or two-way ANOVA (panels E and F) and Tukey’s multiple range tests

Hypermethylation of the promoter region leading to inactivation of oncogenes is one of the common features shared by human tumors. To investigate whether the abnormally reduced expression of MYH11 in GC is associated with hypermethylated modifications in the promoter region, we first obtained the sequence of the CpG island located in the MYH11 promoter from NCBI (Fig. 6A). The correctness of the CpG island sequence was confirmed by MethPrimer (Fig. 6B). Subsequently, the methylation level of MYH11 promoter in GC tumor tissues and adjacent tissues was detected by qMSP (Fig. 6C). We observed a significant elevation of MYH11 promoter methylation levels in tumor tissues. Similarly, the methylation level of MYH11 promoter was significantly augmented in GC cells compared to GES-1 cells (Fig. 6D). We treated GC cells with the DNA methylation inhibitor 5-aZa-CDR, which successfully inhibited the methylation of MYH11 promoter in GC cells as detected by qMSP (Fig. 6E). The expression of MYH11 was significantly elevated in GC cells after inhibition of MYH11 promoter methylation by RT-qPCR (Fig. 6F). It was shown that the reduction of MYH11 expression in GC depends on the DNA hypermethylation of its promoter region.

### DNMT3B induces DNA hypermethylation of MYH11 promoter

**Fig. 7:**
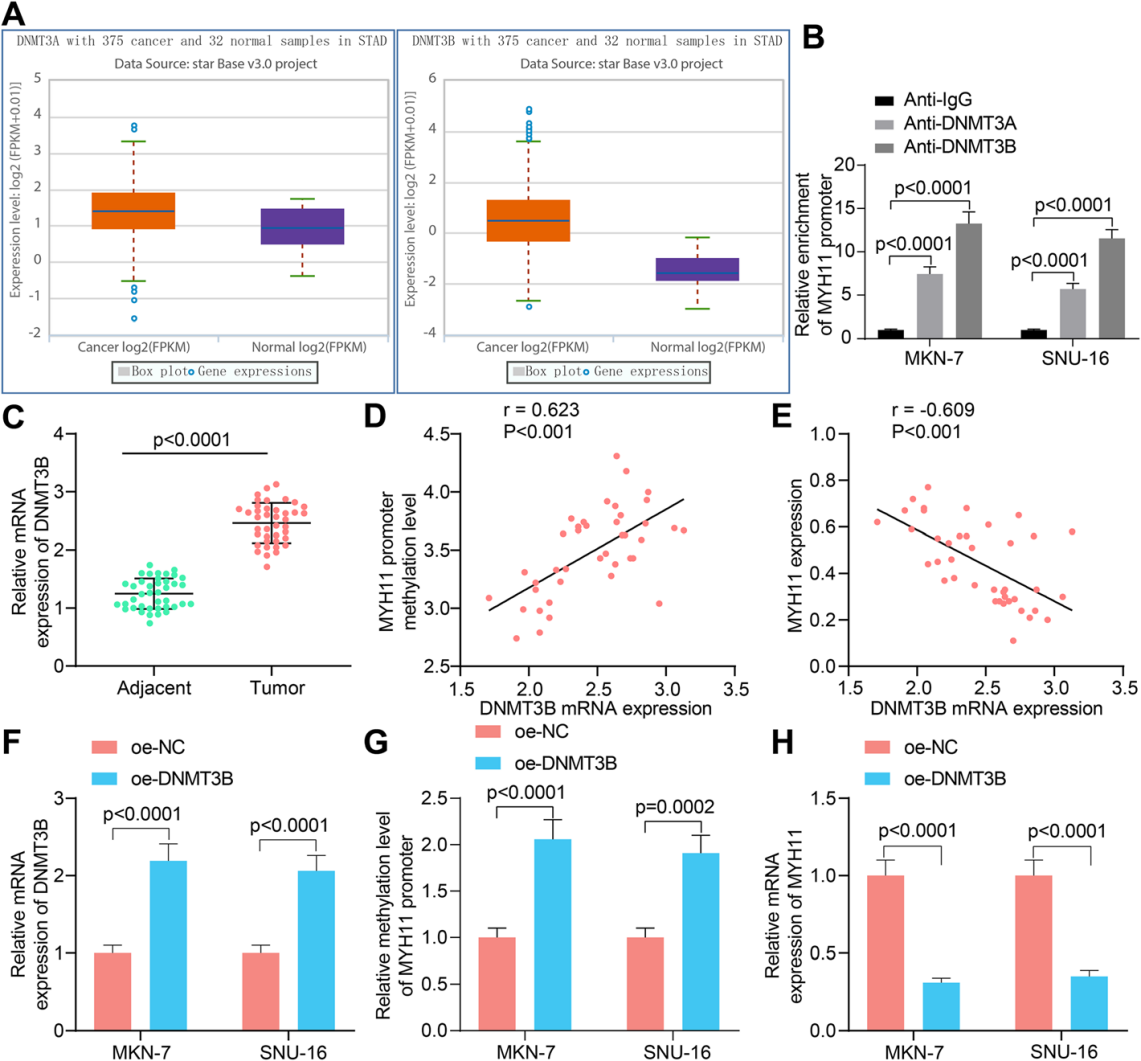
DNMT3B upregulation is responsible for the hypermethylation of the MYH11 promoter. **A**, the expression of DNMT3A and DNMT3B in GC was queried in StarBase Pan-cancer platform; **B**, the enrichment ability of DNMT3A and DNMT3B on MYH11 promoter examined by ChIP-qPCR; **C**, DNMT3B mRNA expression in GC tumor tissues and their adjacent tissues by RT-qPCR; **D**, correlation of DNMT3B expression with MYH11 promoter methylation levels in tumor tissues analyzed by Pearson’s correlation analysis (r = 0.623, *p* < 0.001); **E**, correlation of DNMT3B expression with MYH11 expression in tumor tissues (r = − 0.609, *p* < 0.001); **F**, transfection efficiency of oe-DNMT3B in GC cells by RT-qPCR; **G**, the effects of DNMT3B overexpression on the methylation level of MYH11 promoter examined by qMSP; **H**, effects of DNMT3B overexpression on MYH11 expression by RT-qPCR. Each assay was performed at least three times. Statistical significance was analyzed by paired *t* test (panel C) or two-way ANOVA (panels B, F, G and H) and Tukey’s multiple range tests

We queried the expression profile of DNA methyltransferases DNMT3A and DNMT3B, which have been described to be related to CpG island methylation in GC, in the StarBase Pan-cancer platform (http://starbase.sysu.edu.cn/panCancer.php), and we observed that the expression of both was elevated in GC (Fig. 7A). The enrichment ability of DNMT3A and DNMT3B for MYH11 promoter was examined by ChIP-qPCR, and we observed that both could enrich MYH11 promoter sequence, while DNMT3B showed more prominent enrichment ability (Fig. 7B). Therefore, we selected DNMT3B for the following study.

The expression of DNMT3B was detected by RT-qPCR in the collected tumor tissues and adjacent tissues, and we found that its expression was significantly elevated in the tumor tissues (Fig. 7C). The expression of DNMT3B in tumor tissues was positively correlated with the methylation of MYH11 promoter (Fig. 7D) and conversely correlated with the expression of MYH11 (Fig. 7E). We transfected DNMT3B overexpression plasmids into GC cells, and effective transfection was obtained by RT-qPCR (Fig. 7F). qMSP assayed the methylation level of MYH11 promoter in cells, and we observed that overexpression of DNMT3B significantly promoted the methylation modification of MYH11 promoter (Fig. 7G). Further RT-qPCR results also showed that overexpression of DNMT3B suppressed the expression of MYH11 (Fig. 7H). We demonstrated experimentally that MYH11 promoter hypermethylation in GC was caused by the overexpression of DNMT3B.

### Overexpression of MYH11 inhibits tumor growth in vivo

**Fig. 8:**
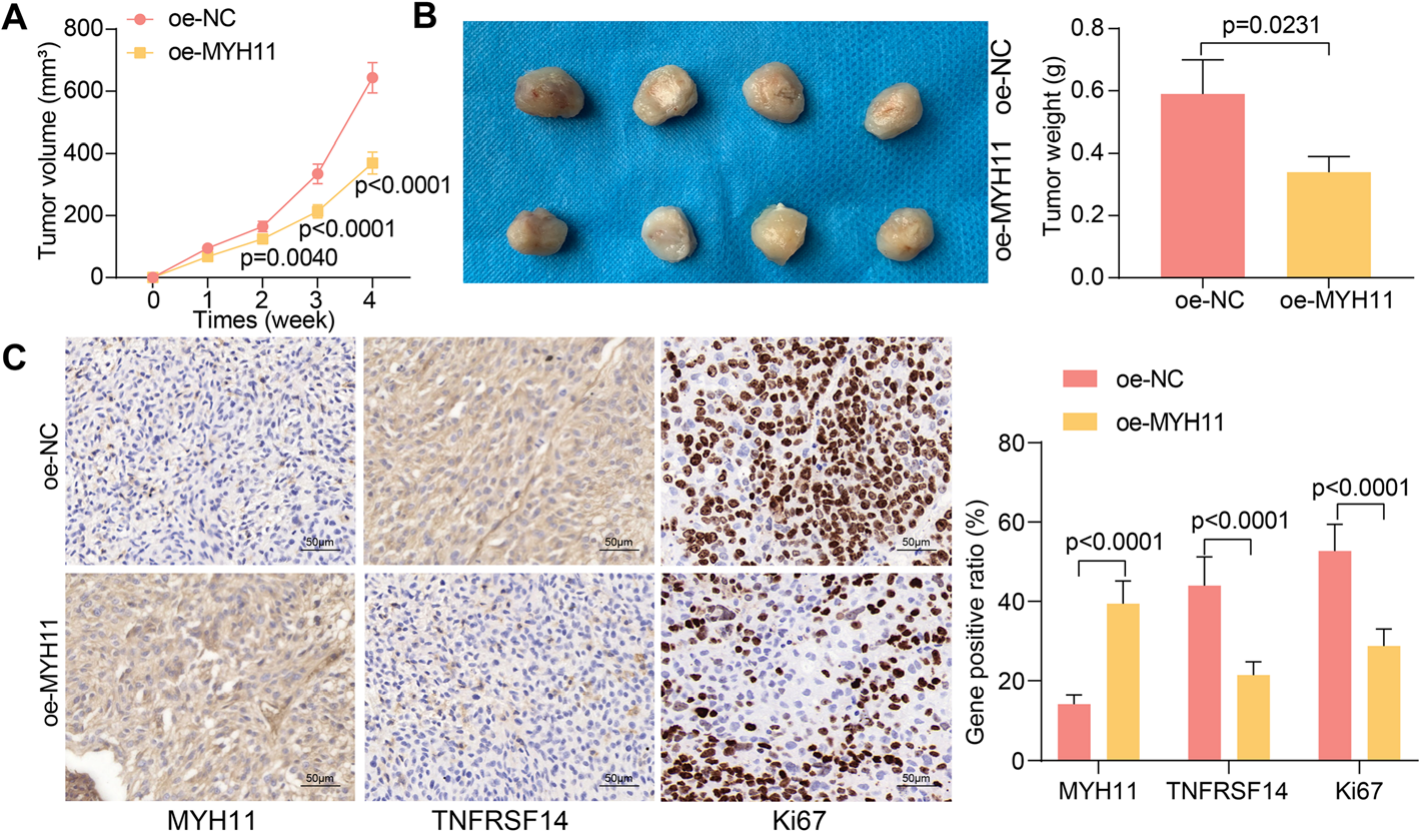
MYH11 overexpression impedes tumor growth in GC. GC cells transfected with oe-MYH11 or oe-NC were delivered into nude mice (*n* = 4). **A**, tumor growth rate; **B**, weight of harvested xenograft tumors; **C**, immunohistochemical detection of MYH11, TNFRSF14, and Ki67 expression in xenograft tumors. Statistical significance was analyzed by unpaired *t* test (panel B) or two-way ANOVA (panels A and C) and Tukey’s multiple range tests

We injected GC cells overexpressing MYH11 subcutaneously into the ventral side of nude mice to perform tumor xenograft experiments. To reduce unnecessary animal sacrifice, we only used MKN-7 cells for in vivo experiments. Tumor volume was measured weekly for 4 weeks starting on the seventh day of injection. We found that tumor growth was significantly slower in the oe-MYH11 group compared to the oe-NC group (Fig. 8A). Four weeks later, tumor tissues were harvested and weighed (Fig. 8B). A significant decrease was noted in tumor weight in the MYH11 overexpression group. By immunohistochemical detection of MYH11, TNFRSF14, and Ki67 expression in harvested xenograft tumors, we observed a significant increase in MYH11 expression and a significant decrease in Ki67 and TNFRSF14 expression in tumor tissues from the mice injected with cells overexpressing MYH11 (Fig. 8C).

## Discussion

Carcinogenesis of GC is a multistep process characterized by a broad spectrum of molecular alterations [16]. DNA methylation, the most frequent epigenetic mechanism, is spotted almost entirely in CpG dinucleotides which tend to cluster in areas called CpG islands and exists in approximately 70% of human gene promoters [17]. In this study, a relationship was substantiated between MYH11 downregulation and the hypermethylation of its promoter in GC, and poorly expressed MYH11 was correlated with a poor prognosis and was intimately intertwined with tumor size, TNM stage, and lymph node metastasis of GC patients. Additionally, as implied by gain-of-function assays, MYH11 overexpression inhibited cell migration, proliferation and invasion. Therefore, our findings unveiled the tumor suppressor role of MYH11 in GC.

Saeki et al. has long established that MYH11 is not expressed in GC cell lines [18]. Furthermore, colorectal cancer patients with lower MYH11 expression inclined to have a dismal prognosis, and poor MYH11 expression evidenced to be an independent adverse prognosticator [19]. These findings were largely in agreement with the observations of our study. Moreover, MYH11, increased by treatment of H_2_O_2_, could reduce proliferation and elevate apoptosis of mouse aortic smooth muscle cells [20]. While the functional role of MYH11 is less studied in cancers. Our gain-of-function assays exhibited that MYH11 showed anti-proliferatory, anti-migratory, anti-invasive, while pro-apoptotic properties in GC cells. Furthermore, we utilized bioinformatics websites to screen for the downstream target of MYH11. TNFRSF14 (also termed as HVEM) was revealed as the only result. As we mentioned previously, MYH11 could serve as a transcription factor. Therefore, we downloaded the possible binding sites between MYH11 and TNFRSF14 for connection validation. Subsequent ChIP and luciferase reporter assay attested our hypothesis. TNFRSF14 was significantly promoted in non-small cell lung cancer patients with N2 lymph node metastasis or more advanced stage [21]. Overexpression of TNFRSF14 was associated with poor overall survival and disease-free survival in patients with clear cell renal cell carcinoma, and TNFRSF14 silencing resulted in an evident decline in cell growth both in vitro and in vivo [22]. Similarly, Migita et al. reported that knockdown of TNFRSF14 significantly repressed esophageal squamous cell carcinoma cell proliferation in vitro and growth in vivo [23]. More relevantly, the elevated TNFRSF14 levels correlated with the development and poor prognosis of GC [24]. The rescue experiments carried out in the present study demonstrated that upregulation of TNFRSF14 reversed the inhibitory effects of MYH11 overexpression on GC cell malignant phenotype.

Epigenetic alterations, including DNA methylation, might initiate and support changes that contribute to the inactivation of tumor-suppressor and other cancer-associated genes in GC [25]. Figueroa et al. proposed that established CBFb-MYH11 leukemia entity was highly linked to methylation profiles [26]. Thus, we next probed whether the downregulation of MYH11 in GC was induced by the methylation of its promoter as well. qMSP revealed the significant increase in the methylation level of MYH11 promoter in GC tissues and cell lines relative to their counterparts. Additional qMSP and RT-qPCR assays using 5-aZa-CDR further verified that the high methylation level was responsible for the downregulation of MYH11 in GC. Likewise, MSP implemented by Shahmohamadnejad et al. showed that the hypermethylation of miR-124 promoter region led to its remarkable downregulation in colorectal cancer tissues, and miR-124 expression was restored following 5-aZa-CDR treatment [27]. Our ensuing ChIP-qPCR assay determined that DNMT3B, instead of DNMT3A, showed higher enrichment ability for MYH11 promoter sequence. Protein and mRNA expression of DNMT3B were found enhanced in melanoma and GC [28–30]. Also, DNA hypermethylation at the CpG sites in the promoter of N-myc downstream regulated gene 2 observed in GC was related to upregulated DNMT3B expression caused by *H. pylori* treatment [31].

## Conclusion

**Fig. 9:**
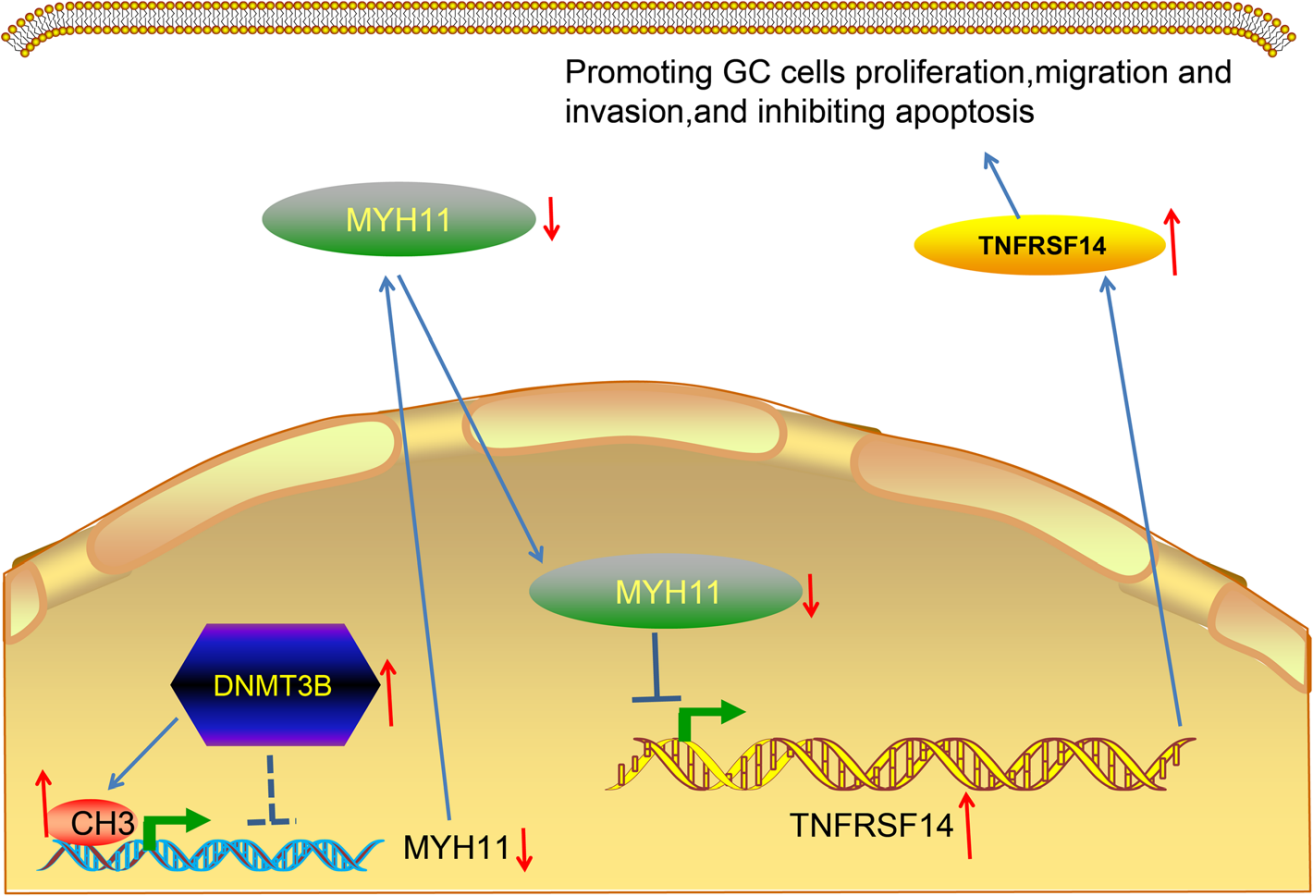
Mechanism diagram. Overexpression of DNMT3B in GC inhibited MYH11 expression by promoting methylation of the MYH11 promoter, thereby attenuating the repressive effect of MYH11 on TNFRSF14 transcriptional activity and promoting GC progression

Overall, we discovered in this study that MYH11 expression is markedly decreased in GC tissues and cells, and that the loss of MYH11 expression is correlated with an unsatisfactory prognosis of GC patients. Overexpression of MYH11 increased the apoptosis of GC cells, and reduced the growth and tumorigenicity of GC cells by reducing the expression of TNFRSF14. Furthermore, our results indicated that DNMT3B exerts an inhibitory effect on MYH11 expression by methylation of its promoter (Fig. 9). These results suggested that MYH11 may be a crucial prognostic and therapeutic target for patients with GC, providing a new direct for future research.

## Supplementary Information

**Additional file 1.**

## Data Availability

All the data generated or analyzed during this study are included in this published article.

## Abbreviations

CCK-8: cell counting kit-8
ChIP: chromatin immunoprecipitation
DAPI: 4′,6-diamidino-2-phenylindole
DNMT3B: DNA methyltransferase 3b
FBS: fetal bovine serum
GAPDH: glyceraldehyde-3-phosphate dehydrogenase
GC: gastric cancer
MSP: Methylation specific PCR
MYH11: myosin heavy chain 11
NC: negative control
OD: optical density
TNFRSF14/HVEM: herpes virus entry mediator
TUNEL: Terminal deoxynucleotidyl transferase (TdT)-mediated 2′-Deoxyuridine 5′-Triphosphate (dUTP) nick end labeling
