# Supplementary figures and images for: Interaction between DNMT3B and MYH11 via hypermethylation regulates gastric cancer progression
Source: BMC Cancer. 2021 Aug 12;21:914. doi: 10.1186/s12885-021-08653-3 (PMC8359574; doi:10.1186/s12885-021-08653-3)

**Fig 1F**

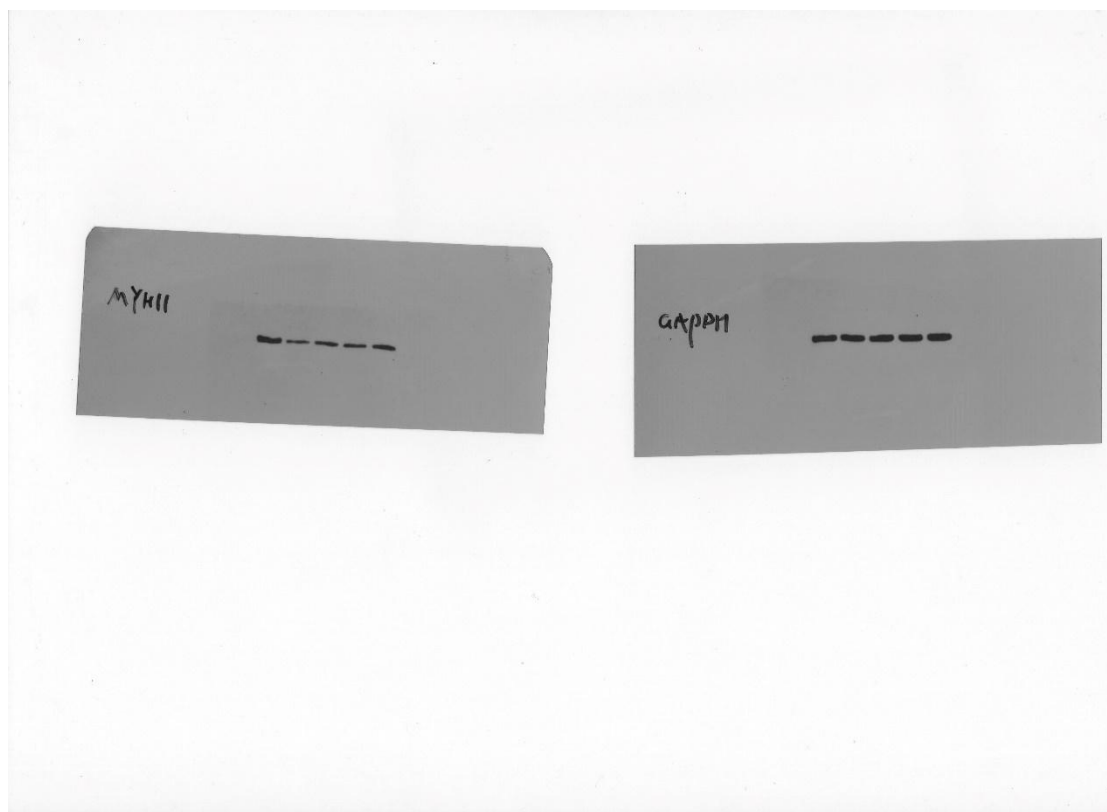

**Fig 3G**

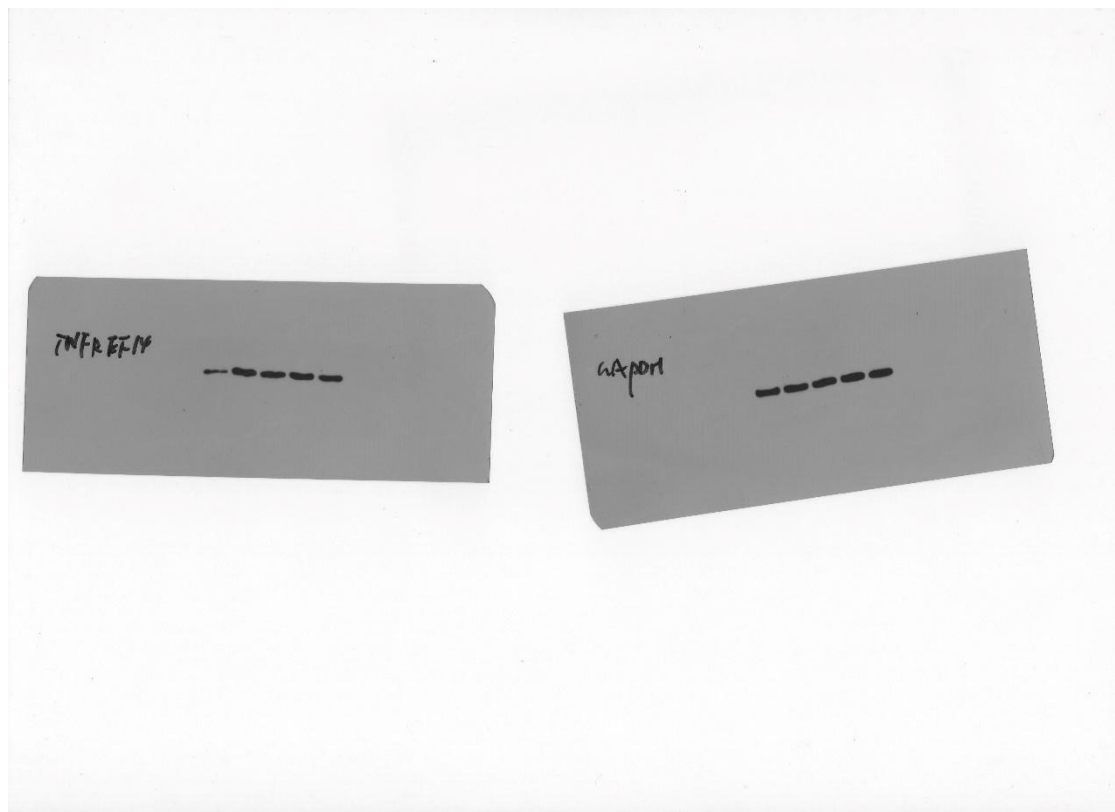

**Fig 4B**

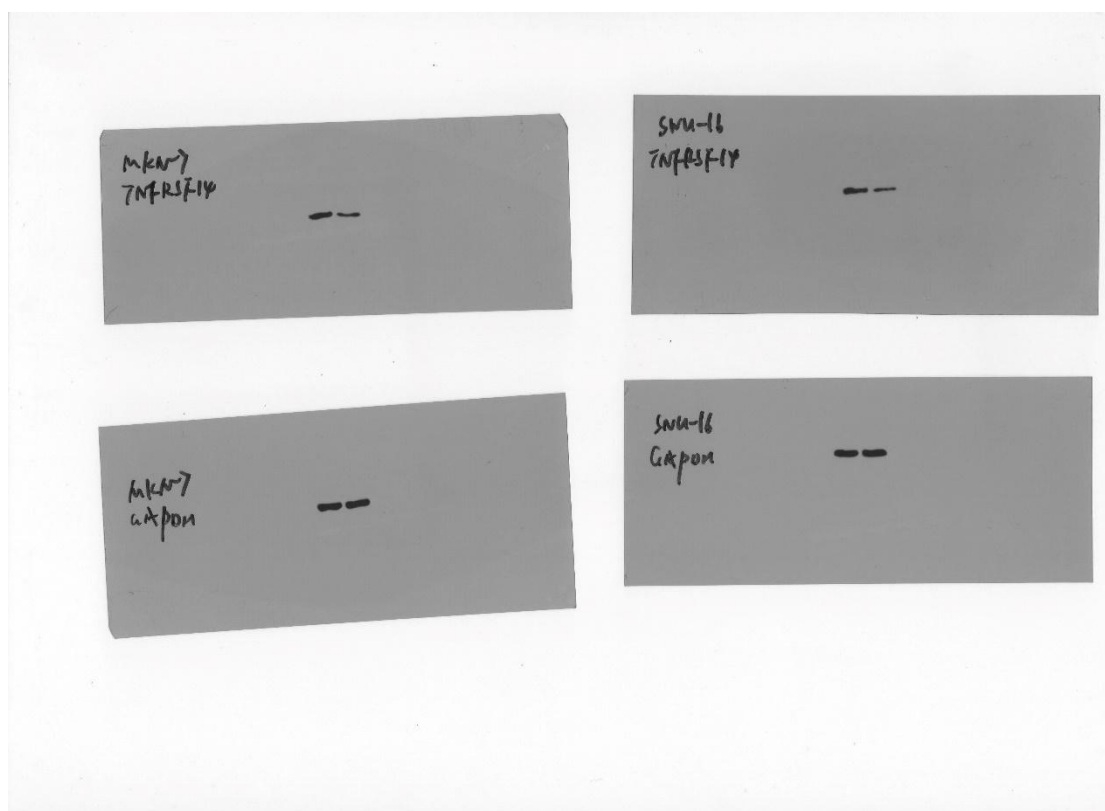

Supplement: Supplementary file 1 — Additional file 1. [file 12885_2021_8653_MOESM1_ESM.pdf]
